# Dual mTORC1/2 inhibition induces anti-proliferative effect in NF1-associated plexiform neurofibroma and malignant peripheral nerve sheath tumor cells

**DOI:** 10.18632/oncotarget.7099

**Published:** 2016-01-31

**Authors:** Jennifer Varin, Laury Poulain, Mikael Hivelin, Patrick Nusbaum, Arnaud Hubas, Ingrid Laurendeau, Laurent Lantieri, Pierre Wolkenstein, Michel Vidaud, Eric Pasmant, Nicolas Chapuis, Béatrice Parfait

**Affiliations:** ^1^ EA7331, Faculté de Pharmacie de Paris, Université Paris Descartes, Sorbonne Paris Cité, Paris, France; ^2^ Institut Cochin, Département d'Immuno-Hématologie, CNRS UMR8104, INSERM U1016, Paris, France; ^3^ Service de Chirurgie Plastique et Reconstructrice, Hôpital Européen Georges Pompidou- AP-HP, Université Paris Descartes, Paris, France; ^4^ Service de Biochimie et de Génétique Moléculaire, Hôpital Cochin, AP-HP, Paris, France; ^5^ Département de Dermatologie, Centre de Référence des Neurofibromatoses, Hôpital Henri-Mondor, AP-HP, Créteil, France; ^6^ EA 4393 LIC, Université Paris Est Créteil (UPEC), Créteil, France; ^7^ Service d'Hématologie Biologique, Hôpital Cochin, AP-HP, Paris, France

**Keywords:** dual mTORC1/2 inhibitor, NF1, MPNST, plexiform neurofibromas

## Abstract

Approximately 30-50% of individuals with Neurofibromatosis type 1 develop benign peripheral nerve sheath tumors, called plexiform neurofibromas (PNFs). PNFs can undergo malignant transformation to highly metastatic malignant peripheral nerve sheath tumors (MPNSTs) in 5-10% of NF1 patients, with poor prognosis. No effective systemic therapy is currently available for unresectable tumors. In tumors, the *NF1* gene deficiency leads to Ras hyperactivation causing the subsequent activation of the AKT/mTOR and Raf/MEK/ERK pathways and inducing multiple cellular responses including cell proliferation. In this study, three *NF1*-null MPNST-derived cell lines (90-8, 88-14 and 96-2), STS26T sporadic MPNST cell line and PNF-derived primary Schwann cells were used to test responses to AZD8055, an ATP-competitive “active-site” mTOR inhibitor. In contrast to rapamycin treatment which only partially affected mTORC1 signaling, AZD8055 induced a strong inhibition of mTORC1 and mTORC2 signaling in MPNST-derived cell lines and PNF-derived Schwann cells. AZD8055 induced full blockade of mTORC1 leading to an efficient decrease of global protein synthesis. A higher cytotoxic effect was observed with AZD8055 compared to rapamycin in the *NF1*-null MPNST-derived cell lines with IC50 ranging from 70 to 140 nM and antiproliferative effect was confirmed in PNF-derived Schwann cells. Cell migration was impaired by AZD8055 treatment and cell cycle analysis showed a G0/G1 arrest. Combined effects of AZD8055 and PD0325901 MEK inhibitor as well as BRD4 (BromoDomain-containing protein 4) inhibitors showed a synergistic antiproliferative effect. These data suggest that NF1-associated peripheral nerve sheath tumors are an ideal target for AZD8055 as a single molecule or in combined therapies.

## INTRODUCTION

Neurofibromatosis type 1 (NF1; Mendelian Inheritance in Man [MIM] ID 162200) is an autosomal disorder with a worldwide birth incidence of one in 2500. Approximately 30-50% of individuals with NF1 develop benign peripheral nerve sheath tumors, called plexiform neurofibromas (PNFs) that are usually congenital or appear in early childhood [1, 2]. PNFs are complex tumors, heterogeneous at the cellular level, mainly composed of Schwann cells, which are the likely pathogenic cell type in neurofibromas, together with fibroblasts, mast cells, neurons, vascular elements, and perineurial cells [3]. Although benign, PNFs are often life threatening by their proximity to internal organs and can undermine quality of life [3]. Surgery is only palliative for most patients, and face allotransplantation has been proposed for patients with massive PNFs [4]. PNFs can undergo malignant transformation to malignant peripheral nerve sheath tumors (MPNSTs) in 5-10% of NF1 patients [5]. MPNSTs are highly metastatic with a poor vital prognosis. The poor response to available systemic treatment modalities underscores the need for more effective and targeted therapies in the treatment of NF1-associated peripheral nerve tumors.

NF1 is caused by dominant loss-of-function mutations of the tumor suppressor gene *Neurofibromin 1* (*NF1*) encoding neurofibromin, a negative regulator of RAS (*rat sarcoma*) proteins. *NF1* loss of heterozygosity was found in Schwann cells isolated from PNFs and MPNSTs [6, 7]. Loss of *NF1* can therefore enhance RAS activation and promote signaling down to two main pathways: the mitogen-activated protein kinase (MAPK) pathway and the AKT/mTOR pathway.

RAF kinase becomes active upon binding to RAS-GTP and initiates the MEK/ERK phosphorylation cascade, leading to increases in gene transcription that promote cell growth and survival. A specific pharmacological inhibitor of MEK1 and MEK2 (called PD0325901) was shown to induce a tumor growth decrease and a prolonged survival in a human MPNST xenograft model [8].

The mTOR kinase controls intracellular mechanisms like cell growth, proliferation and survival. mTOR is a serine/threonine kinase that belongs to the phospho-inositide 3-kinase (PI3K)-related kinase family and is ubiquitously expressed in mammalian cells. mTOR resides in at least two distinctive multi-protein complexes, mTORC1 and mTORC2, which are distinguished by their partner proteins, their substrate specificities and their differential sensitivity to rapamycin; mTORC1 regulates protein synthesis by activating the ribosomal protein S6 Kinase (P70S6K) and inactivating the eukaryotic initiation factor 4E (eIF4E)-binding proteins (4E-BPs). In contrast, the role of mTORC2 has only recently emerged in cancer cell biology and is mainly related to the control of AKT Ser^473^ phosphorylation. The mTOR inhibitor rapamycin (sirolimus) was shown to suppress the growth of NF1-associated malignancies in a genetically engineered murine model [9]. However, rapamycin only binds mTORC1 *via* FKBP12 protein binding and in most of cases does not inhibit the mTORC2 complex that plays a key role in cellular survival and proliferation by up-regulating AKT.

Clinical trials using pharmacological agents targeting RAS-MAPK pathways (including MEK inhibitors) and AKT/mTORC1 pathways (rapamycin and rapalogs) are currently under evaluation for PNFs (http://www.clinicaltrials.gov/ct2/results?term=nf1) [10, 11]. In previous preclinical studies using NF1-tumor mouse models, both MEK and mTORC1 inhibitors showed tumors growth suppression properties but no cytolytic effect. Different mechanisms underlying resistance to rapamycin have been described and could explain this moderate activity: (i) the rapamycin-induced increase of PI3K activity, (ii) the lack of complete mTORC1 inhibition as attested by the sustained high level of 4E-BP1 phosphorylation, and (iii) the inefficiency of rapamycin towards mTORC2 activity.

Recently, loss-of-function mutations of the histone-modifying complex polycomb repressive complex 2 (PRC2) were described in MPNSTs [12, 13]. PRC2 loss led to increased levels of acetylated histone H3 of lysine 27 (H3K27Ac), which recruits bromodomain proteins [14]. MPNST cell lines were shown to be sensitive to bromodomain inhibitors [12, 15].

In the present study, we tested a new ATP-competitive “active-site” mTOR inhibitor AZD8055 that directly suppresses the mTOR catalytic activity in human NF1-associated MPNST cell lines and plexiform neurofibromas derived primary Schwann cells. Contrary to rapamycin, we demonstrate that AZD8055 inhibited the activity of both mTORC1 and mTORC2, resulting to an important decrease of cell growth and proliferation by blocking cell cycle progression. Combined targeting of the PI3K/AKT/mTOR pathway with the dual mTORC1 and mTORC2 inhibitor, AZD8055 and the MAPK pathway with the MEK inhibitor, PD0325901 was effective to synergistically inhibit cell growth in NF1-associated MPNST and NF1-derived Primary Schwann cells. For the first time, we also demonstrated that AZD8055 and BET bromodomain proteins inhibitors exert a synergistic cell growth inhibitor effect in MPNST cell lines. Together, these data suggest that AZD8055 or AZD8055-based combination therapies may comprise a novel and efficacious therapy for patients harboring NF1-associated peripheral nerve sheath tumors.

## RESULTS

### *NF1* genotyping in MPNST cell lines and PNF-derived primary Schwann cells

MPNST cell line 90-8 presented a hemizygous 7bp deletion in exon 23-1 (c.3904_3910delGATCCTT, NM_000267.3 = *p*.(Asp1302fs) NP_000258.1), as previously reported [16]. MPNST cell line 88-14 presented an hemizygous 1bp insertion in exon 11 (c.1649dupT = *p*.(Val551fs)). MPNST cell line 96-2 presented a homozygous 1bp deletion in exon 21 (c.3684delC = *p*.(Ala1228fs)). We confirmed the complete *NF1* locus heterozygous deletion previously reported in the STS26T MPNST cell line [17]. PNF-derived primary Schwann cells and paired peripheral blood leukocytes were also genotyped. A constitutional *NF1* mutation was identified in leukocyte DNAs for 8/8 patients and a somatic inactivation of the *NF1* wild-type allele was identified in 7/8 of the corresponding PNF-derived primary Schwann cells DNAs with *NF1* locus loss-of-heterozygosity (LOH) in 6/7 cases (Table 1).

**Table 1:** PNF-derived primary Schwann cells NF1 genotyping

	Germline event	Somatic event
PNF1	c.4267A>G = p.Lys1423Glu	LOH
PNF2	c.204+1G>A	LOH
PNF3	c.1095_1096delAA = p.Arg366fs	IVS3113+1G>AG
PNF4	c.1541_1542delAG = p.Gln514fs	Copy Neutral LOH
PNF5	c.480-2A>G	Copy Neutral LOH
PNF6	c.4903delA = p.Thr1635fs	Copy Neutral LOH
PNF7	c.8135dupA = p.Lys2712fs	Copy Neutral LOH
PNF8	c.4999G>T = p.Glu1667*	Not identified

A *NF1* heterozygous germline mutation was identified in peripheral blood leukocytes DNA in 8/8 patients. A *NF1* somatic event was identified in DNA extracted from 7/8 PNF-derived primary Schwann cells.

### Molecular characterization MPNST cell lines and PNF-derived primary Schwann cells

Genome-wide array-CGH was used to identify potential genetic rearrangements in MPNST cell lines and PNF-derived primary Schwann cells (Supplemental Figure S1). We confirmed that STS26T, 90-8, 88-14, and 96-2 MPNST cell lines have rearranged genomes [17–18]. Evidence for deletions of the *NF1* locus were found in DNAs from 3/4 of the MPNST cell lines (STS26T, 90-8, and 88-14), as previously described (Supplemental Figure S1) [17–18]. Deletions at locus 9p21.3 (including the *CDKN2A*/*B* locus) were found in 4/4 of the MPNST cell lines, in accordance with previous data showing that > 80% of MPNSTs presented somatic alterations of *CDKN2A/B* [12].

Evidence for deletions of the *NF1* locus were found in DNAs from 3/8 of the PNF-derived primary Schwann cells: PNF1, PNF2, and PNF8 (Table 1; Supplemental Figure S1). A deletion spanning chromosome region 9p21.3 was identified in 1/8 of the PNF-derived primary Schwann cells (PNF8), in accordance with previous data describing recurrent deletions at locus 9p21 in NF1-associated PNFs [19–20]. No other chromosome alterations were found in the 8 PNF-derived primary Schwann cells.

### AZD8055 induced a strong inhibition of mTORC1 and mTORC2 signaling in MPNSTs-derived cell lines

**Figure 1:**
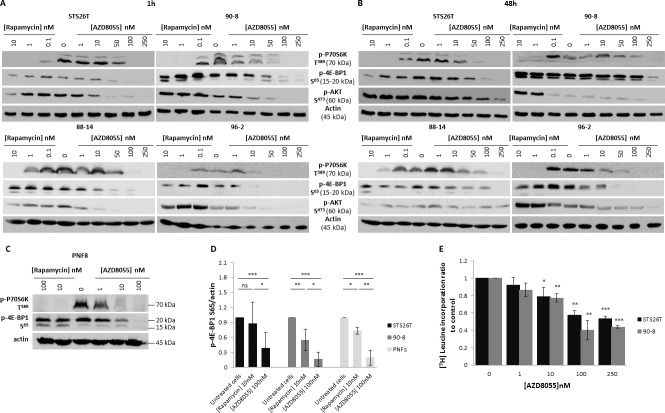
AZD8055 strongly blocks both mTORC1 and mTORC2 signaling pathways and inhibits protein translation in MPNST cell lines **A.** STS26T, 90-8, 88-14 and 96-2 MPNST cell lines were cultured 1h without or with crescent rapamycin (0.1 to 10 nM) or AZD8055 (1 to 250nM) concentrations. mTORC1 and mTORC2 activities were assessed by Western blots using p-P70S6K T^389^, p-4E-BP1 S^65^ and p-AKT S^473^. **B.** STS26T, 90-8 88-14, 96-2 MPNST cell lines cells were cultured 48h without or with crescent rapamycin (0.1 to 10 nM) or AZD8055 (1 to 250nM) concentrations. mTORC1 and mTORC2 activities were assessed by Western blots using p-P70S6K T^389^, p-4E-BP1 S^65^ and p-AKT S^473^. **C.** Primary Schwann cells from 4 patients were cultured 1h without or with crescent rapamycin (10 to 100 nM) or AZD8055 (1 to 100 nM) concentrations. mTORC1 and mTORC2 activities were assessed by Western blots using p-P70S6K T^389^, phospho-4E-BP1 S^65^ and p-AKT S^473^. **D.** p-4E-BP1 S^65^ was quantified and normalized to actin signal intensity in 3 independent experiments in 90-8 and STS26T cell lines and in primary Schwann cells from 4 patients (2 patients for experiments with rapamycin). Results are expressed as a ratio to the control incubation without AZD8055 (100nM) or rapamycin (10nM). **E.** [^3^H]leucine pulses were performed to determine global protein synthesis rates in 90-8 and STS267 cells cultured with or without AZD8055 (1 to 250 nM). Results are presented as the ratio to the control incubation without inhibitor.

The biochemical activity of the mTOR kinase inhibitor AZD8055 against mTORC1 and mTORC2 signaling was first tested in STS26T, 90-8, 88-14 and 96-2 MPNST cell lines and compared to the mTORC1 allosteric inhibitor, rapamycin. P70S6K T^389^ phosphorylation, reflecting mTORC1 activity, was efficiently inhibited after 1 hour of exposure to either rapamycin or AZD8055 (Figure 1A). In all tested cell lines, rapamycin failed to inhibit the phosphorylation of the translation regulator 4E-BP1 on S^65^, thereby reflecting an incomplete effect on mTORC1 signaling. In contrast, AZD8055 induced a strong inhibition of p-4E-BP1 S^65^ from 10, 50 or 100nM depending on tested cell lines. The quantification of Western Blot signals obtained from three independent experiments showed a decrease of p-4E-BP1 S^65^ of 84% *vs*. 45% when using AZD8055 (100nM) or rapamycin (10nM) respectively in 90.8 cell lines, and a decrease of 82% *vs* 12% when using AZD8055 (100nM) or rapamycin (10nM) respectively in STS26T cell lines (Figure 1D). Similar results were also observed after 48h exposure to rapamycin or AZD8055 (Figure 1B).

Also, we compared the direct activity of AZD8055 *vs*. rapamycin towards mTORC2 signaling. AKT S^473^ phosphorylation, reflecting mTORC2 activity was not decreased with 1h of rapamycin exposure, confirming that the allosteric inhibition of mTORC1 does not repress mTORC2 activity (Figure 1A). At 48h, in STS26T, 90-8, and 96-2 cell lines, AKT phosphorylation on S^473^ was even increased, probably due to the inhibition of the P70S6K-induced negative feedback loop on PI3K/AKT signaling, (Figure 1B) [21, 22]. In contrast, AZD8055 inhibited AKT phosphorylation on S^473^ after 1h exposure indicating that this compound represses mTORC2 activity in MPNST cell lines.

Activity of AZD8055 was also tested in primary Schwann cells. Rapamycin only partially blocked mTORC1 signaling as attested by the full suppression of p-P70S6K T^389^ and the lack of p-4E-BP1 S^65^ decrease. Conversely, AZD8055 strongly inhibited mTORC1, attested by both p-P70S6K T^389^ and p-4E-BP1 S^65^ inhibition, and mTORC2, reflected by p-AKT S^473^ suppression (Figure 1C). The quantification of Western blot signals obtained from 4 tested primary Schwann cells samples confirmed the difference in rapamycin and AZD8055 efficacy on mTORC1 signaling (decrease of 66% and 19.5% when using 100nM AZD8055 and 10nM rapamycin respectively) (Figure 1D).

### AZD8055 inhibited protein synthesis in MPNST cell lines

The phosphorylation of 4E-BP1 is the limiting step in the assembly of the translation initiating complex eIF4F, initiated by the interaction between eIF4E and eIF4G [23]. AZD8055 could inactivate protein translation through 4E-BP1 S^65^ phosphorylation blockade. We performed [^3^H] leucine assays, in which the detection of radioactivity is proportional to the amounts of neo-synthesized proteins. A concentration-dependent decrease in protein synthesis was observed and became significant with 100nM AZD8055 (60% and 42% for 90-8 and STS26T cell lines respectively). This inhibition correlates to the concentration of AZD8055 required to fully inhibit the phosphorylation of 4E-BP1 on S^65^ in all cell lines tested (Figure 1E).

### AZD8055 decreased proliferation in MPNST-derived cell lines and PNF-derived primary Schwann cells

**Figure 2:**
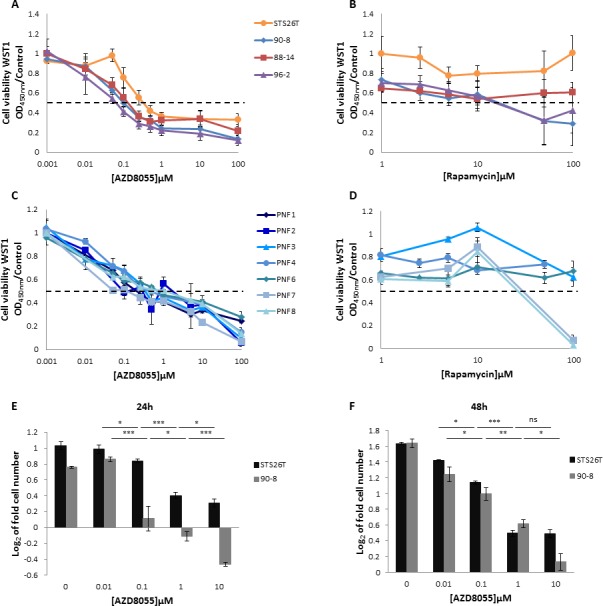
AZD8055 impairs cell lines proliferation Cells were treated with increasing doses of AZD8055 or rapamycin for 48 hours. The dose response curves on cell growth were assessed by using WST1 assay. Effects on cell viability are expressed as a function of μM drug concentrations (log scale). **A.** Dose response curves of MPNST-derived cell lines treated with AZD8055 or **B.** rapamycin. **C.** Dose response curves of PNF-derived primary Schwann cells treated with AZD8055 or **D.** rapamycin.**E.** Cytostatic and cytotoxic AZD8055 effect was observed for STS26T and *NF1*-null 90-8 cell lines respectively when cultured with different concentrations of AZD8055 during 24 hours. Cells were counted and log_2_ (T24 cell number/T0 cell number) was calculated. **F.** A dose-dependent cytostatic effect was more pronounced for *NF1*-null 90-8 cell line when cultured with 10μM of AZD8055 during 48 hours. Cells were counted and log_2_ (T48 cell number/T0 cell number) was calculated.

The dose-response analysis after 48 hours of continuous exposure of AZD8055 or rapamycin was first performed on the four MPNST-derived cell cultures. The three *NF1*-null MPNST-derived cell lines were sensitive to AZD8055 with IC50_AZD8055_ values of 107±30nM, 137±36nM and 67±10nM for 90-8, 88-14 and 96-2 cell lines, respectively. The STS26T sporadic MPNST cell line was less sensitive to AZD8055 with an IC50_AZD8055_ value of 335±97nM (Figure 2A). MPNST cell lines 96-2 and 90-8 were sensitive to high doses of rapamycin with IC50_rapamycin_ values of 11±5.6μM and 11.7±5μM, respectively. STS26T and 88-14 did not respond to rapamycin treatment (Figure 2B). Seven PNF-derived primary Schwann cells were also treated with AZD8055 or rapamycin. AZD8055 IC50_AZD8055_ values were between 100 nM to 1μM (Figure 2C) whereas rapamycin antiproliferative effects where observed only for 2/5 PNF-derived primary Schwann cell when treated with 50μM (Figure 2D).

We also performed 90-8 *vs*. STS26T cell counting in presence of AZD8055. After 24h, a short term dose dependent cytotoxic effect was observed in 90-8 MPNST-derived cell line from 1μM AZD8055 treatment (Figure 2E). To a lower extent, a dose dependent decrease of STS26T proliferation was also observed without reaching cytotoxicity. After 48h of treatment, AZD8055 induced a dose dependent decrease of both 90-8 and STS26T proliferation. Cytostatic effect was reached at a dose of 10μM for 90-8 cell line only (Figure 2F).

### AZD8055 decreased MPNST-derived cell lines migration

**Figure 3:**
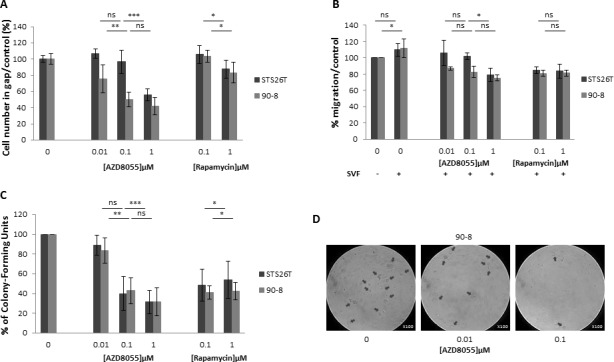
AZD8055 impairs cell migration and anchorage independent growth in soft agar **A.** Cell lines migration behavior was studied with IBIDI culture-inserts. Subconfluent cells were treated with AZD8055 or rapamycin. After 6 hours of treatment, each well was photographed and cells were counted. Percentage of treated cells *versus* untreated cells in gap was assessed. **B.** Similar results were obtained when migration behavior was studied using a transwell assay. AZD8055 significantly reduced the migratory ability of STS26T and 90-8 MPNST-derived cell migration with a dose dependent response. **C.** Quantification of soft agar assay performed with 90-8 cell lines treated with 0, 0.01, 0.1, 1 μM AZD8055 or 0.1, 1 μM rapamycin. **D.** Representative microscopy observations of 90-8 cell line in soft agar after 8 days of AZD8055 treatment (x100).

Cell migration was examined by wound-healing assays and Boyden transwell system after AZD8055 or rapamycin treatment. STS26T and 90-8 MPNST-derived cell migration to a standardized 500μm gap was significantly decreased with a dose dependent response from 0.1μM to 1μM AZD8055 during 6h (Figure 3A), 24 and 48h (Supplemental Figure S2). This result was confirmed with Boyden transwell chambers (Figure 3B).

### AZD8055 decreased the *in vitro* tumorigenicity of MPNST-derived cell lines

The anchorage-independent growth in soft agar was assessed and results indicated a significant colony number and size decrease after AZD8055 as well as rapamycin treatment for both STS26T and 90-8 cell lines in comparison with untreated cell lines (Figure 3C and 3D).

### AZD8055 impaired cell cycle progression in MPNST-derived cell lines

**Figure 4:**
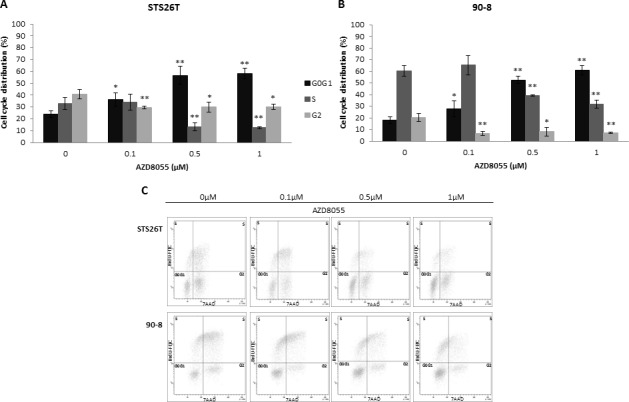
AZD8055 impairs cell cycle progression in STS2T and 90-8 cell lines Cell cycle analysis was performed on STS26T and 90-8 cell lines after 24 hours exposure to different doses of AZD8055 The dose-dependent G0/G1 phase increase and S phase reduction is observed for both **A.** STS26T and **B.** 90-8 cell lines. **C.** A representative experiment (*n* = 3) is also presented.

To explore the AZD8055 cell growth inhibition mechanism, apoptosis/necrosis and cell cycle analyzes were performed in MPNST-derived cell lines using flow cytometry. Apoptosis/necrosis was not significantly induced by AZD8055 treatment (data not shown). In contrast, cell cycle progression was impaired. After 24h of treatment, AZD8055 significantly inhibited MPNST-derived cell lines proliferation *via* inducing G0/G1 phase arrest supported by S and G2 phase decrease combined to G0/G1 phase increase in both 90-8 and STS26T cell lines (Figure 4).

### Dual blockade of both mTOR and MAPK pathways was synergistic in MPNST cell lines and PNF-derived primary Schwann cells

**Figure 5:**
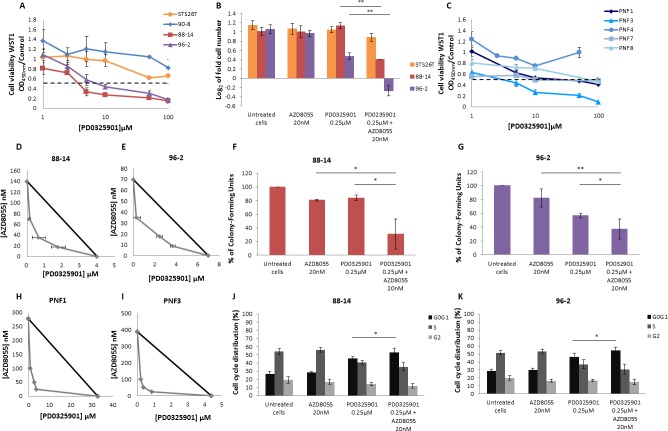
AZD8055 combined with PD0325901 impairs synergically cell lines proliferation and cell cycle progression. Cells were treated with increasing doses of PD0325901 for 48 hours The dose effect curves on cell growth were assessed by using WST1 assay. Effects on cell viability are expressed as a function of μM drug concentrations (log scale). **A.** Dose response curves of MPNST-derived cell lines treated with PD0325901 were assessed. **B.** Cells were counted and log_2_ (T48 cell number/T0 cell number) was calculated. Cytostatic and cytotoxic effect was observed for *NF1*-null 88-14 and 96-2 cell lines respectively when cultured with AZD8055 combined to PD0325901 during 48 hours. **C.** Dose response analysis of PNF-derived primary Schwann cells were also performed with increasing doses of PD0325901 for 48 hours. **D.** and **E.** Combination isobolograms using the combination AZD8055/PD0325901 were assessed. The IC50 values of PD0325901 and AZD8055 were plotted on the × and y axes along with the IC50 values of PD0325901 obtained in the presence of various fixed concentrations of AZD8055. The diagonal line drawn between the IC50 values for the two molecules on the y and × axes is the theoretical line of additivity. All IC50 values to the left of this line indicate synergy. A synergistic effect was observed when *NF1*-null 88-14 and 96-2 cell lines. **F.** and **G.** Analysis of attachment-independent growth in soft agar was performed and revealed that AZD8055 combined to PD0325901 synergistically decreased 88-14 and 96-2 tumorigenic potential. **H.** and **I.** Combination isobolograms showing a synergistic effect of AZD8055/PD0325901 combination for PNF-derived primary Schwann cells. **J.** and **K.** AZD8055 combined with PD0325901 synergistically impaired cell cycle progression for 88-14 and 96-2 cell lines. These experiments were realized three times.

We first performed dose-response analysis after 48 hours of continuous exposure of the PD0325901 MEK inhibitor on MPNT-derived cell lines and five PNF-derived primary Schwann cells. 88-14 and 96-2 were sensitive (IC50_PD901_ = 3.7±0.8μM and IC50_PD901_ = 6.8±2.3μM, respectively) whereas STS26T and 90-8 were not sensitive (Figure 5A). PD0325901 response was also assessed on PNF-derived primary Schwann cells showing variable sensitivity. IC50_PD901_ were assessed for PNF1 and PNF3 at 33 and 3.8μM, respectively (Figure 5C).

Then, we performed 88-14 and 96-2 *vs*. STS26T cell counting in presence of AZD8055 and PD0325901 to investigate the combined antiproliferative effect. The no-effect 20nM dose of AZD8055 was combined to 0.25μM PD0325901 MEK inhibitor. This latest dose showed little to any effect on cell proliferation when PD0325901 was used as a single molecule on 88-14 and 96-2 cell lines. When the two molecules were used in combination during 48 hours, no effect was observed on STS26T control cell line compared to AZD8055 treated cells. Conversely, a significant synergy was observed on 88-14 and 96-2 cell lines with a cytotoxic response for 96-2 (Figure 5B). A synergy was also observed on tumorigenicity abilities of both cell lines (Figure 5F, 5G).

AZD8055 combined to PD0325901 synergistic effect was also investigated on 88-14, 96-2 MPNST cell lines, PNF1 and PNF3 PNF-derived primary Schwann cells by using combination isobolograms. We performed dose response analysis of PD0325901 in combination with fixed AZD8055 doses and IC50 values were calculated. IC50_AZD8055_ and IC50_PD901_ of each cell lines were plotted on AZD8055/PD0325091 combination isobolograms with arbitrarily IC50/2, IC50/4 and IC50/8 AZD8055 dose settings for MPNST cell lines and 100, 50 and 25nM dose settings for PNF-derived primary Schwann cells. Combination isobolograms clearly revealed AZD8055/PD0325091 synergy at all explored concentrations in MPNST cell lines and in PNF-derived primary Schwann cells (Figure 5D, 5E, 5H and 5I).

To explore AZD8055 and PD0325901 synergistic cell growth inhibition, cell cycle distribution analysis by flow cytometry was performed. Neither 88-14 nor 96-2 cell lines cell cycle was impaired when cells were treated with the no-effect 20nM dose of AZD8055 (Figure 5J and 5K). In contrast, cell cycle was affected in both cell lines when treated with 0.25μM PD0325901 with an increase in G0/G1 phase and reduction in S phase. However when both treatments were combined, a significant induced G0/G1 phase arrest was observed compared to PD0325901 used as a single molecule showing the synergy of the two molecules.

### Dual blockade of both mTOR pathway and chromatin structure regulation was synergistic in MPNST cell lines

**Figure 6:**
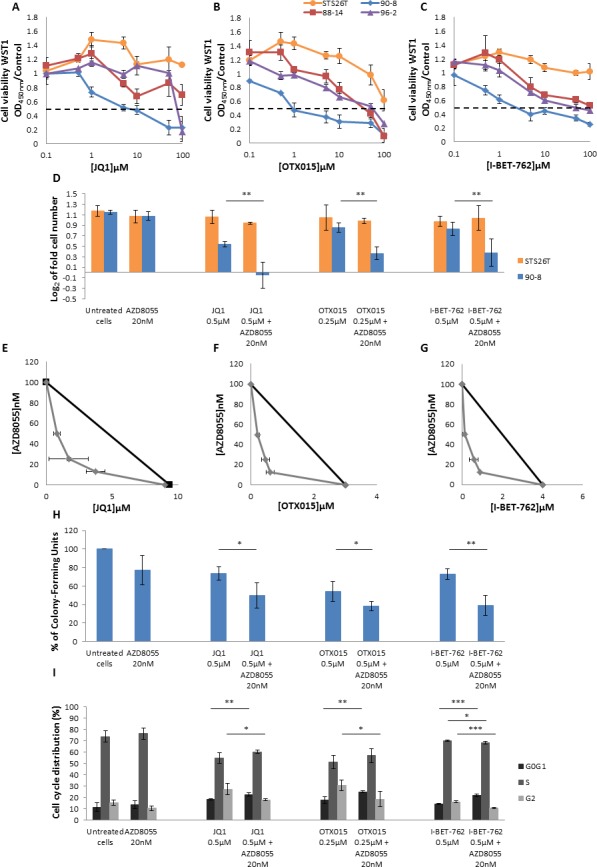
AZD8055 and bromodomain inhibitors have a synergic effect on MPNST-derived cell lines proliferation and cell cycle progression **A.**, **B.** and **C.** Dose response curves of MPNST-derived cell lines treated with three bromodomain inhibitors: JQ1, OTX015 and I-BET-762. **D.** Synergistic effect was observed for *NF1*-null 90-8 cell line when cultured with AZD8055 combined to each of the three bromodomain inhibitors during 48 hours. Cells were counted and log_2_ (T48 cell number/T0 cell number) was calculated. **E.**, **F.**, **G.** Combination isobolograms using the combinations AZD8055/bromodomains inhibitors were assessed. A synergistic effect was observed when *NF1*-null 90-8 cell line was treated with AZD8055 combined to each of the three bromodomain inhibitors. **H.** Analysis of attachment-independent growth in soft agar was performed and revealed that AZD8055 combined to bromodomain inhibitors synergistically decreased 90-8 tumorigenic potential. **I.** AZD8055 and JQ1, OTX015 or I-BET-762 combined treatment synergistically impaired cell cycle progression for the 90-8 cell line. These experiments were repeated three times.

Dose-response analyzes after 48 hours of continuous exposure of the three JQ1, OTX015, I-BET-762/GSK525762 BET bromodomain inhibitors were performed in STS26T, 90-8, 88-14, and 96-2 MPNST-derived cell lines. 90-8 cell line was sensitive to JQ1 (IC50_JQ1_ = 9±5μM). To a lesser extent, 96-2 cell line was also sensitive to JQ1 (IC50_JQ1_ = 66±22μM). In contrast, 88-14 and STS26T cell lines were JQ1-resistant (Figure 6A). 90-8 cell line was sensitive to OTX015 (IC50_OTX015_ = 3.1±0.3μM) and, to a lesser extent, 88-14 and 96-2 cell lines were also (IC50_OTX015_ = 46±6μM and IC50_OTX015_ = 55±9μM, respectively). In contrast, STS26T was OTX015-resistant (Figure 6B). 90-8 cell line was sensitive to I-BET-762 (IC50_I-BET-762_ = 3.8±0.25μM) and, to a lesser extent, 96-2 and 88-14 cell lines were also (IC50_I-BET-762_ = 66±32μM and IC50_I-BET-762_ = 95±10μM respectively). In contrast, STS26T was I-BET-762-resistant (Figure 6C). IC50_JQ1_ were assessed for PNF1 derived primary Schwann cells (IC50_JQ1_ = 55μM, *n* = 1). PNF3 derived primary Schwann cells did not response to JQ1 (not shown). Due to little or no response of PNF-derived primary Schwann cells to JQ1, IC50 were only assessed with JQ1 BET bromodomain inhibitor.

AZD8055 and BET bromodomain proteins inhibitors combined antiproliferative effect was investigated in 90-8 cell line, as 90-8 cell line was JQ1/OTX015/I-BET-762 sensitive. We performed 90-8 cell counting in presence of AZD8055 and each of BET bromodomain inhibitors as single molecules and in combined treatment. Doses of bromodomain inhibitors where chosen because they showed only slight effect on cell proliferation when used as a single agent. When the no-effect 20nM dose of AZD8055 was used in combination with any of the three BET bromodomain inhibitors during 48 hours, a significant synergistic effect was observed on 90-8 cell line with a cytostatic response to JQ1 (Figure 6D). No effect was observed on STS26T control cell line compared to AZD8055 treated cells.

Moreover, combination isobolograms clearly confirmed AZD8055/BET bromodomain inhibitors synergy at all explored concentrations in 90-8 cell line (Figure 6E, 6F and 6G). Soft agar colony formation assay also confirmed a synergistic decrease of the cell line tumorigenic potential (Figure 6H).

When compared to untreated cells, 90-8 cell cycle was not impaired by 20nM AZD8055 treatment (Figure 6I). However, cell cycle was impaired when 90-8 cells were treated with JQ1 or OTX015 with S phase decrease coupled with G0/G1 and G2 phases increase. When cells were treated with AZD8055 combined to JQ1 or OTX015, significant G0/G1 increase and G2 decrease was observed. The 0.5μM I-BET-762 treatment did not impair 90-8 cell cycle. However, combined to 20nM AZD8055, we observed a significant synergistic G0/G1 increase coupled to G2 decrease (*p* value < 0.001) supporting the relevance of combined mTOR kinase inhibitor AZD8055 and BET bromodomain inhibitors therapies. To a lesser extent, 96-2 cell line was also sensitive to BET bromodomain inhibitors and we confirmed the synergistic effect of AZD8055 combined to BET bromodomain inhibitors on this second cell line (Supplemental Figure S3).

## DISCUSSION

Upregulation of the PI3K pathway, in particular, has been demonstrated as a key contributor to tumor progression in many cancer types [24]. The abnormal activation of the RAS/MEK pathway also occurs in many cancers and significant crosstalk with the PI3K pathway can occur [25]. In previous studies, mTOR inhibition by rapamycin or rapalogs (everolimus *alias* RAD001) showed antitumor activity on MPNST cell lines *in vitro*. mTOR inhibitors have been both characterized as leading to cell cycle arrest by suppressing cyclin D1 expression, inducing apoptosis, and inhibiting cell proliferation and angiogenesis, and shown to repress cell motility and invasion [9, 21, 26, 27]. It was suggested that rapamycin or rapalogs can induce paradoxical AKT activation due to a negative feedback loop between p70S6K and the insulin-like receptor substrate-1 and that they also can enhance MAPK pathway signaling, leading to resistance to rapamycin or rapalogs [21, 22, 28].

In this study, we tested an ATP-competitive “active-site” mTOR inhibitor that directly suppresses the mTOR catalytic activity [29, 30]. We showed that a subset of NF1-associated peripheral nerve sheath tumors cells dependent on RAF/MEK/ERK and PI3K/AKT/mTOR signaling for growth was exquisitely sensitive to the clinically available dual TORC1/C2 inhibitor AZD8055, as a single agent. AZD8055 induced a strong inhibition of mTORC1 and mTORC2 signaling in MPNSTs-derived cell lines and PNF-derived primary Schwann cells. Interestingly, complete inhibition of mTORC1 activity leads to a strong decrease of protein translation in MPNST cell lines. We then compared the efficacy of AZD8055 *versus* rapamycin (an allosteric inhibitor of mTORC1). Our results confirmed the limitations of rapamycin treatment of MPNST as rapamycin failed to block mTORC2 activity. Furthermore, rapamycin induced only partial inhibition of mTORC1 signaling which correlates with inefficiency on protein translation (data not shown). We also showed that AZD8055 decreased proliferation in MPNST-derived cell lines *via* inducing G0/G1 phase arrest. These results provide the rational to directly target the mTOR catalytic activity with AZD8055 to alleviate these intrinsic mechanisms of resistance in NF1-associated PNFs and MPNSTs.

Due to *NF1* loss in MPNST, RAS proteins are activated. Inhibitions of MAPK and mTOR pathways have been evaluated in MPNST preclinical studies. As single agents, no tested inhibitor caused tumor regression, revealing the need for combinatorial approaches in MPNST [2, 8, 9, 21, 26, 27, 31].

Crosstalk between the MAPK and AKT/mTOR pathways is well documented. MEK inhibitors induce epidermal growth factor-induced AKT activation, ERK phosphorylates TSC2 and RAPTOR to promote TORC1 activity, and AKT can phosphorylate RAF at inhibitory sites that negatively regulate MEK activity [32]. Co-targeting the MAPK and AKT/mTOR pathways has been evaluated, raising high hopes for MPNST treatment [33]. The highly selective pharmacological inhibitor of MEK, PD0325901 has been tested in preclinical trials as a single molecule or in combination with rapamycin or rapalogs. Here, we show that AZD8055 can cooperate with anti-MEK PD0325901 to promote tumor cells regression in two distinct NF1-associated peripheral nerve sheath *in vitro* 2D and 3D models. We also show AZD8055/PD0325901 combination efficacy *ex vivo* using PNF-derived primary Schwann cells and we clearly demonstrated the synergistic effect. Therefore, dual blockade of both mTOR and MAPK pathways offers a new hope of treatment for PNF growth thus preventing malignant progression to MPNST and for MPNST.

Recently, we and other reported the role of Polycomb repressive complex 2 (PRC2) loss-of-function mutations in the MPNST pathogenesis [12, 34]. PRC2 inactivation potentiates the effects of *NF1* mutations by amplifying Ras-driven transcription through effects on chromatin. PRC2 inactivation also triggers an epigenetic switch from H3K37Me3 to H3K27Ac. Histone acetylation/deacetylation status is a crucial epigenetic regulation of gene expression and addition of acetyl groups to histone lysine residues by histone acetyl transferase turns the chromatin to an active transcriptional state [35]. The bromodomain and extra-terminal (BET) family (BRD2, BRD3, BRD4 and BRDT) function as readers of the acetylated chromatin. Deregulation of these proteins is found in several diseases including cancer [35]. Therefore, they are attractive potential therapeutic targets for new anticancer drug development. The BromoDomain-containing protein 4 (BRD4) binds H3K27Ac and recruits the positive transcriptional elongation factor b, p-TEFb complex to the chromatin and activates transcription by phosphorylation of RNA polymerase II [36]. BRD4 is a known regulator of G1/S transition in mouse fibroblasts where it activates transcription of Cyclin D1 and other genes involved in cell cycle progression [37]. BRD4 is upregulated in MPNSTs and has an important role in maintaining tumorigenic capacity of NF1-associated MPNSTs *in vivo*. BRD4 inhibition has been shown to suppress growth and tumorigenesis [38]. Moreover, MPNST-derived cell line 90-8 is known to respond to JQ1 bromodomain inhibitor [12]. In this study, we performed dose response analyzes on four MPNST-derived cell lines using JQ1 and two other bromodomain inhibitors OTX015 and GSK525762A/I-BET762. JQ1 (thieno-triazolo-1,4-diazepine) competitively binds to the acetyl-lysine recognition site of BRD4 and subsequently prevents BRD4 recruitment to acetylated histone of the chromatin leading to transcription repression [39]. OTX015 is an oral JQ1 analog. Its pharmacological effects have recently been described in acute myeloid and lymphoid leukemias and this molecule is currently used in a phase I-II trial in relapsed/refractory leukemia patients [40, 41]. GSK525762A/I-BET762 belongs to the quinolone class of BET inhibitors and is also orally used [42]. This molecule is also currently evaluated in phase I-II clinical trials in relapsed/refractory leukemia and NUT Midline Carcinoma (http://www.clinicaltrials.gov/ct2/results?term=nf1). We observed that these three therapeutic agents used as single molecules impaired cell proliferation and induced G0/G1 phase increase especially in MPNST-derived cell line 90-8 in which PRC2 is known to be inactivated [12]. To a lesser extent 88-14 and 96-2 cell lines showed a slight response to OTX015 and GSK525762A/I-BET762 compounds. Combined to AZD8055, JQ1, OTX015 and GSK525762A/I-BET762 inhibited 90-8 cell proliferation, soft agar colony formation and impaired cell cycle progression in a synergistic manner thus supporting that dual blockade of both PI3K pathway and chromatin structure regulation is also of interest in MPNST pharmacological treatment.

Based on 2D and 3D *in vitro* results, AZD8055/PD0325901 or AZD8055/BET bromodomain inhibitors combination efficacy now needs to be empirically tested in rigorous models *in vivo* to support these combination strategy for NF1-associated nerve sheath tumors [43].

## MATERIALS AND METHODS

### MPNST cell lines

The MPNST-derived cell lines STS26T, 90-8, 88-14, and 96-2 were maintained in advanced RPMI-1640 with 15% heat-inactivated FBS, 2.75‰ Bovine Pituitary Extract, 100U/ml penicillin, and 100μg/ml streptomycin (Invitrogen). MPNST cell line STS26T was established from a sporadic MPNST and was kindly provided by G. H. De Vries (Hines VA Hospital, Illinois, USA) [44]. MPNST cell lines 90-8, 88-14 and 96-2 were established from NF1-associated MPNST: 90-8 and 88-14 were kindly provided by Pr. N. Ratner (Cincinnati Children's Hospital Medical Center, Ohio, USA) and 96-2 was purchased at ATCC bio resource center [6, 45, 46].

### PNFs-derived primary Schwann cells

Samples were collected in accordance with the French legislation concerning biological samples collection for research (French Ministry of Research, declaration of creation and use of a biological samples collection n° DC-2010-1101 and DC-2012-1604). The study was approved by the local ethical committee (CPP Ile de France III, COL2818) and all patients gave their written informed consent. Blood samples and PNFs tumor resections were obtained from 8 unrelated NF1 patients. Tumors were cut into small pieces, incubated in a standard medium (SM) composed of Dubelcco's modified Eagle's medium DMEM Glutamax-I, 10% heat-inactivated FBS, 100 U/ml penicillin, 100 μg/ml streptomycin (Invitrogen) three to five days and further digested in SM supplemented with 0.125% collagenase (Sigma) and 1.25U/ml dispase grade 1 (Roche) during 20h at 37°C and 9% CO_2_. After a 235G centrifugation, cells were washed twice with SM and suspended in Schwann Cell Medium (SCM) composed of SM supplemented with 0.5 mM 3-iso-butyl-1-methylxanthine (IBMX, Sigma), 10nM β1-heregulin 176-246 (R&D Systems), 0.5μM forskolin (Sigma) and 2.5μg/ml insulin (Sigma). Cells were seeded at 2.6 10^6^ cells per T25 flasks coated with 5 μg/cm^2^ collagen type 1 (Sigma). Cultures were incubated in a humidified atmosphere at 37°C and 9% CO_2_. Twenty four hours after seeding, trypsine 0.002% EDTA (Invitrogen) in PBS was used for cells differential trypsinization. Detaching Schwann cells were collected and seeded in a new T25 flask for a second round of differential trypsinization. Primary Schwann cells were propagated until passage 2 or 3 in SCM at 37°C and 9% CO_2_. Purity of PNF-derived Schwann cells was determined by S100 immunostaining [47]. Only Schwann cell cultures with more than 85% S100 positive cells and not exceeding passage 3 were used for drug assays.

### Microarray procedure and data analysis

Genome-wide array-CGH was used to identify potential genetic rearrangements in MPNST cell lines (400K array-CGH) and PNFs-derived primary Schwann cells (60K array-CGH). The array-CGH labelling and hybridization were performed as recommended in the Agilent manual (Protocol v5.0, June 2007, Agilent technologies, Palo Alto, CA, USA). Each cell line DNA (labelled with Cy5-dUTP) was individually hybridized on Agilent whole human genome 400K or 60K microarrays (Agilent Technologies) *versus* six pooled normal control DNAs (from six healthy donors' blood samples) (labelled with Cy3-dUTP) as reference, to detect cell line-specific aberrations. Arrays were scanned with an Agilent DNA microarray scanner (G2565BA). Log2 ratios were determined with Agilent Feature Extraction software. Results were visualized and analysed with Agilent's CytoGenomics software.

### *NF1* genotyping

DNA was isolated from MPNST-derived cell lines, and eight PNF-derived primary Schwann cell cultures and their leucocytes counterpart using standard proteinase K digestion followed by phenol-chloroform extraction. *NF1* genotyping was then performed using targeted next generation sequencing allowing *NF1* point mutations and copy number variation detection, as previously described [48].

### Tested drugs

AZD8055, JQ1, OTX015, and GSK525762A/I-BET-762 were provided by Selleckchem. Rapamycin and PD0325901 were purchased from Calbiochem. Drugs were dissolved in dimethylsulphoxide (DMSO) to a final stock concentration of 100 mM. Drugs were added in cultures to final working concentrations with no more than 0.1% DMSO.

### Proliferation assays

MPNST cell lines were seeded on 6-well plates at a concentration of 250,000 cells per well in MPNST culture medium. The number of cells was determined (T0) when exponential growing phase was reached. After 24 and 48h of drug treatments, cells were trypsinized and pelleted with corresponding culture supernatant and washing buffers. Viable cells were counted using 0.4% trypan blue solution (Invitrogen). Fold growth was defined as log2 of fold of the number of viable cells treated by different drug concentrations at 24 and 48h *versus* number of cells at T0. Experiments were performed in duplicate and repeated three times.

### *In vitro* cytotoxicity assay

MPNST cell lines and the PNF-derived primary Schwann cell cultures were seeded in triplicate on 96-well plates. Cells were treated with the tested drug or with 0.1% DMSO as negative control. Cytotoxicity was quantified 48 hours after treatment by WST1 assay (Roche). Relative cytotoxicity was determined as ratio of absorbance at 450 nm of treated *versus* untreated cells. IC50 values corresponding to concentrations associated to 50% of cell viability after 48 hours of continuous exposure were determined.

### Migration assay

MPNST cell lines STS26T and 90-8 were seeded at subconfluence on IBIDI culture-inserts as described by manufacturer (Biovalley). After culture-inserts removal, cell migration behavior was tracked after 6, 24 and 48 hours of continuous exposure of increasing doses of AZD8055 and rapamycin. Migration assay was also assessed by using a CytoSelect^TM^ transwell assay as described by the manufacturer (Cell Biolabs). Briefly, 2.5 10^5^ cells/ml in RPMI-1640 with 1% heat-inactivated FBS were preincubated with or without drugs for two hours at 37°C and a 100μl cell suspension was loaded into the upper chamber. RPMI-1640 supplemented with 10% heat-inactivated FBS was used as a chemoattractant in the bottom chamber. After 16 hours of incubation, migrated cells were quantified by fluorimetric analysis as recommended by the manufacturer.

### Soft agar colony formation assay

An acellular basal 0.6% agar layer was plated at the bottom of a 96-well plate and covered with 0.4% agar solution containing 6250 MPNST cell lines. Colonies were quantified by fluorimetric analysis after 8 days of continuous exposure of increasing doses of drugs as described by the manufacturer (Cell Biolabs).

### Cell cycle analysis

MPNST cell lines were serum-starved for 24h and then treated with different drug concentrations. Drug effects on cell cycle were determined by FITC-coupled anti-BrdU antibodies (BD Biosciences) as described by manufacturer (BD Biosciences). Flow cytometry was performed on a BD FACSCanto™ II (BD Biosciences). Results were analysed using the BD FACSDiva™ software version 6.2.1 (BD Biosciences).

### Western blotting

Whole cell extracts and Western blots were performed as previously described [49]. Anti-p-P70S6K T^389^, anti-p-4E-BP1 S^65^, anti-p-AKT S^473^ primary antibodies were purchased from Cell Signaling Technology and anti-actin was purchased from Sigma-Aldrich. Proteins were visualized using a secondary antibody conjugated to horseradish peroxidase and chemiluminescence detection (enhanced chemiluminescence, GE Healthcare). Images were captured using a CCD camera (LAS3000, FujiFilm, Tokyo, Japan) and quantified using Multigauge software (Fujifilm). Images were captured using a CCD camera (LAS3000, Fujifilm)

### [H^3^] Leucine incorporation assay

The global protein synthesis was assessed by [H^3^] Leucine assay. Cells were incubated for 2h at 10^5^ cells/mL in a low leucine medium (90% Dulbecco modified Eagle medium without leucine, 10% MEM with 10% dialysed FCS) then labelled for 1h with [3H] leucine (1 μCi, 37kBq). The amount of radioactivity incorporated into proteins was determined by trichloroacetic acid precipitation.

### Statistical analysis

Data were expressed as mean values and SD. Statistical significance of differences observed between experimental groups was determined using Student's t test. *, **, *** mean *p* < 0.05, *p* < 0.01, *p* < 0.001 respectively.

## SUPPLEMENTARY MATERIAL FIGURES


