# Supplementary material for: Dual mTORC1/2 inhibition induces anti-proliferative effect in NF1-associated plexiform neurofibroma and malignant peripheral nerve sheath tumor cells
Source: Oncotarget. 2016 Jan 31;7(24):35753–67. doi: 10.18632/oncotarget.7099 (PMC5094959; doi:10.18632/oncotarget.7099)
Supplement: Supplementary file 1 [file oncotarget-07-35753-s001.pdf]

Dual mTORC1/2 inhibition induces anti-proliferative effect in NF1-associated plexiform neurofibroma and malignant peripheral nerve sheath tumor cells

Supplementary Material

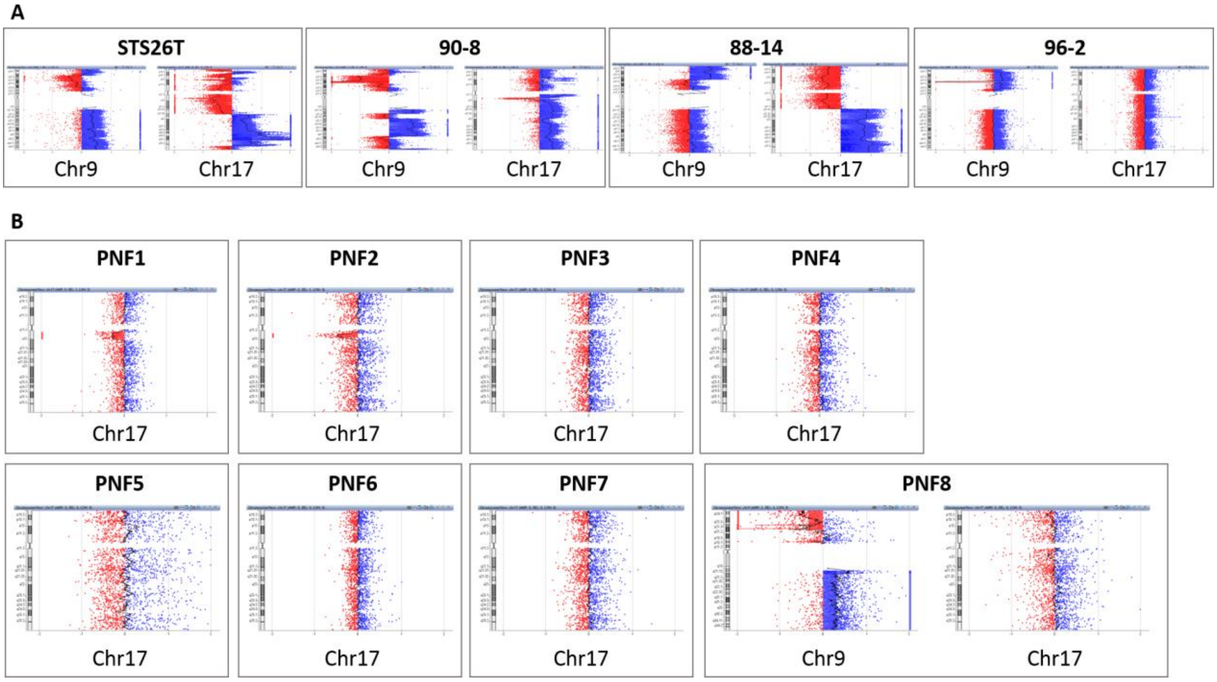

Supplemental Figure S1: Genome-wide array-CGH in MPNST cell lines and PNF-derived primary Schwann cells

A. STS-26T, 90-8, 88-14, and 96-2 cell lines 400K array-CGH chromosomes 9 and 17 profiles. **B.** Primary Schwann cells cell lines 60K array-CGH chromosome 9 (PNF8) and chromosome 17 (PNF1- PNF8) profiles.

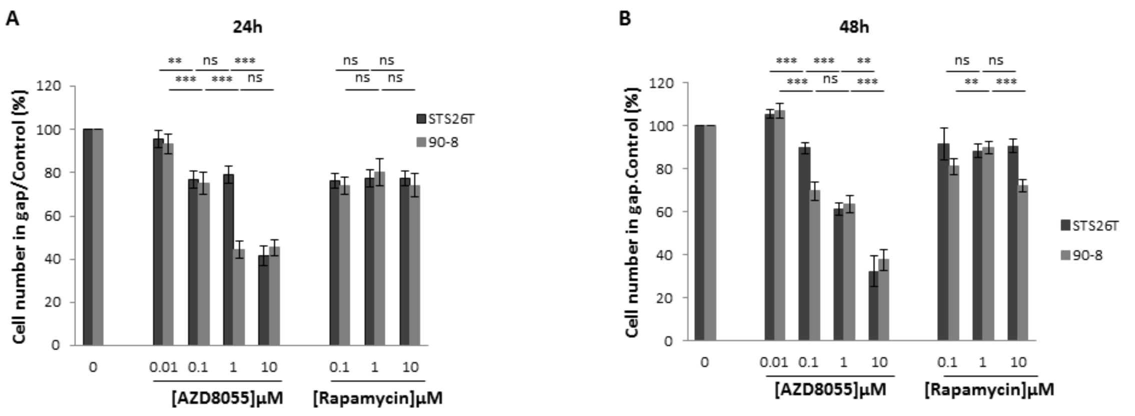

Supplemental Figure S2: AZD8055 impairs cell lines proliferation and migration

A. and **B.** Cell lines migration behavior was studied with IBIDI culture-inserts. Subconfluent cells were also treated with AZD8055 or rapamycin during 24 and 48 hours. Each well was then photographed and cells were counted. Percentage of treated cells *versus* untreated cells in gap was assessed. In contrast to rapamycin, STS26T and 90-8 MPNST-derived cell migration to a standardized 500µm gap was significantly decreased with a dose dependent response to AZD8055 treatment. Cell proliferation was probably also affected by the high-dose AZD8055 treatment (10µM) and the long incubation time (24 to 48 hours).

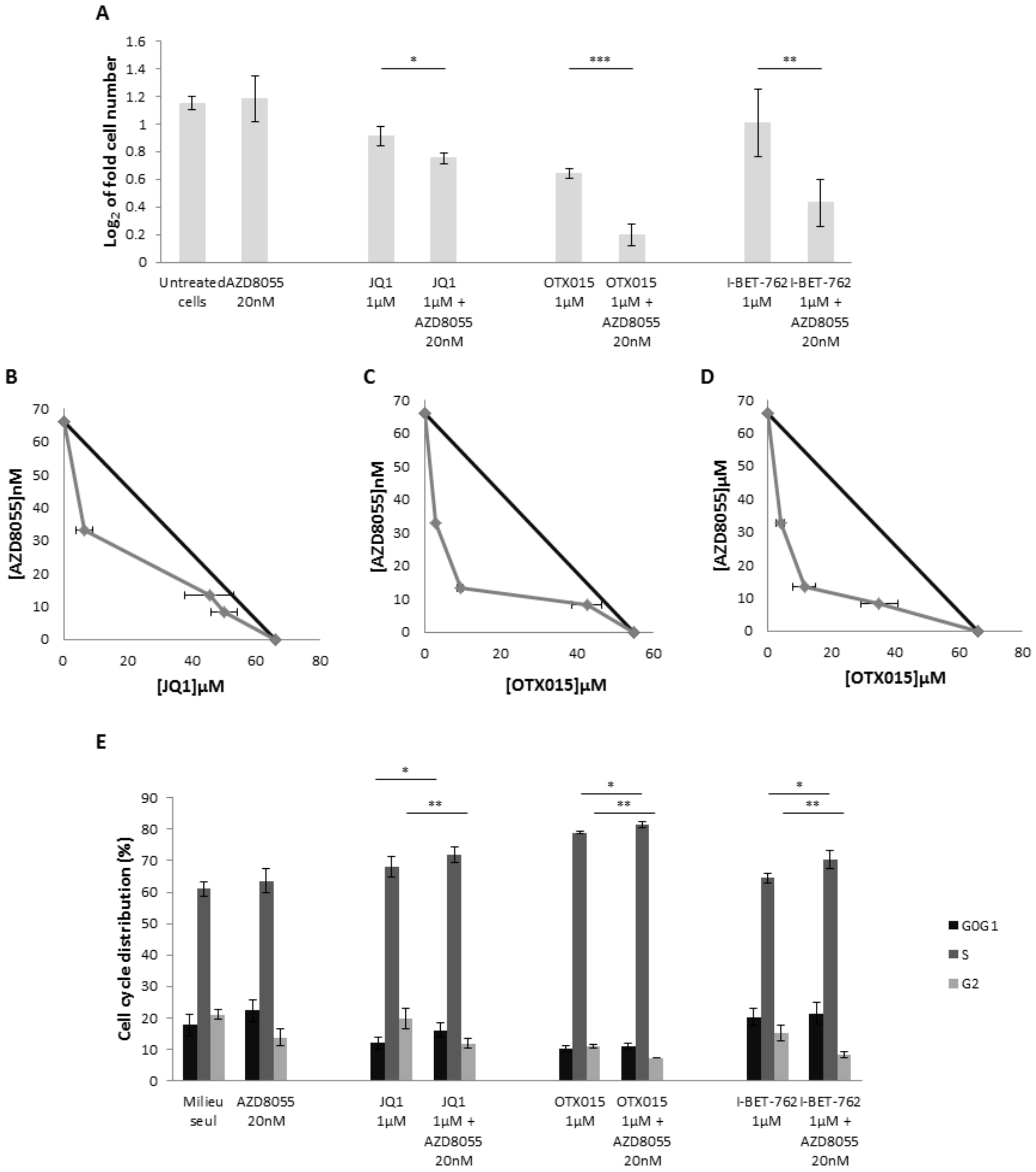

Supplemental Figure S3: AZD8055 and bromodomain inhibitors have a synergic effect on 96-2 MPNST-derived cell line proliferation and cell cycle progression

A. Synergistic effect was observed for *NF1*-null 96-2 cell line when cultured with AZD8055 combined to each of the three bromodomain inhibitors during 48 hours. Cells were counted and $\log_2$ (T48 cell number/T0 cell number) was calculated. **B.**, **C.**, **D.** Combination isobolograms using the combinations AZD8055/bromodomains inhibitors were assessed. A synergistic effect was observed when *NF1*-null 96-2 cell line was treated with AZD8055 combined to each of the three bromodomain inhibitors. **E.** AZD8055 and JQ1, OTX015 or I-

BET-762 combined treatment synergistically impaired cell cycle progression for the 96-2 cell line. These experiments were repeated three times.
